# Hydrogen isotope effect on self-organized electron internal transport barrier criticality and role of radial electric field in toroidal plasmas

**DOI:** 10.1038/s41598-022-09526-w

**Published:** 2022-04-01

**Authors:** T. Kobayashi, A. Shimizu, M. Nishiura, T. Ido, S. Satake, T. Tokuzawa, T. Ii Tsujimura, K. Nagaoka, K. Ida

**Affiliations:** 1 National Institute for Fusion Science, National Institutes of Natural Sciences, Toki, 509-5292 Japan; 2 The Graduate University for Advanced Studies, SOKENDAI, Toki, 509-5292 Japan; 3 Research Institute for Applied Mechanics, Kyushu University, Kasuga, 816-8580 Japan

**Keywords:** Magnetically confined plasmas, Nuclear fusion and fission

## Abstract

Self-organized structure formation in magnetically confined plasmas is one of the most attractive subjects in modern experimental physics. Nonequilibrium media are known to often exhibit phenomena that cannot be predicted by superposition of linear theories. One representative example of such phenomena is the hydrogen isotope effect in fusion plasmas, where the larger the mass of the hydrogen isotope fuel is the better the plasma confinement becomes, contrary to what simple scaling models anticipate. In this article, threshold condition of a plasma structure formation is shown to have a strong hydrogen isotope effect. To investigate the underlying mechanism of this isotope effect, the electrostatic potential is directly measured by a heavy ion beam probe. It is elucidated that the core electrostatic potential transition occurs with less input power normalized by plasma density in plasmas with larger isotope mass across the structure formation. This observation is suggestive of the isotope effect in the radial electric field structure formation.

## Introduction

A long-standing open question in the magnetically confined plasma study is the hydrogen isotope effect^1–5^: the heavier the fuel particles are the better the confinement becomes, contrary to what simple scaling models anticipate. Here, the simple scaling models refer to, e.g., Bohm or gyro-Bohm theory, where diffusion of plasmas produced with heavier hydrogen isotope is expected to be large due to their large diffusion step size^6^. The hydrogen isotope effect has been classically assessed by means of the global confinement time scaling or the local diffusivity. When evaluating such parameters, it is usually inevitable to rely on specific assumptions in heating profile calculation or transport models, which can be sources of uncertainty. Moreover, the hydrogen isotope effect in global or local transport coefficients is not always distinct but can be marginal particularly in stellarators^5^. Therefore, a large scale statistics is necessary for drawing a conclusive trend, which can hamper interpretation on detailed underlying process.

The hydrogen isotope effect is also known to be evident in the edge transport barriers (ETBs)^4,7–9^ or the internal transport barriers (ITBs)^10^, where the threshold condition for the transition is eased in plasmas with larger ion mass. The transport barrier criticality can be qualitatively defined without any model assumption, therefore an experiment-driven discussion for unveiling the underlying physics of the hydrogen isotope effect is possible. Furthermore, as the future thermonuclear fusion plant is currently planned to be operated with the tritium-deuterium mixture fuel and with transport barriers, understanding of the background mechanism of the hydrogen isotope effect is important for projecting the reactor plasma performance. When the transport barriers emerge, a localized radial electric field structure is spontaneously formed by the quasi-neutral plasma^11^, which is believed to suppress the anomalous turbulent transport. Direct measurement of the radial electric field in systematic isotope experiments is therefore a key factor for resolving this issue, but has been challenging for a long time.

In this article, the hydrogen isotope effect on the ITB criticality in the Large Helical Device (LHD) stellarator is elaborated, focusing upon the ITB in the electron temperature profile, the so-called electron ITB. The threshold value in the input power normalized by plasma density for triggering the electron ITB is found to be lowered in plasmas with larger isotope mass. By means of heavy ion beam probe (HIBP), the core electrostatic potential behavior across the ITB formation is investigated. A transition to a positive electrostatic potential, likely leading to a positive radial electric field, occurs with a lower input power normalized by density in plasmas with heavier ions. This observation is suggestive of the isotope effect in the radial electric field structure formation. The self-organized radial electric field criticality having a susceptibility to plasma ion mass can be a missing piece in resolving the hydrogen isotope effect.

## Results

### Experimental set-up

Hydrogen isotope effect in the electron ITB is dynamically investigated by means of an input power modulation experiment in LHD (see Methods for details). In the present experiments, the target plasma is produced by balanced tangential neutral beams (NBs) with $P_{\mathrm{NB}}=3.6$ MW. Electron cyclotron resonance heating (ECH) focused at the torus core is modulated with the peak-to-peak amplitude of $P_{\mathrm{ECH}}=1$ MW and the frequency of $f_{\mathrm{mod}}=23$ Hz. In order to assess the threshold input power normalized by plasma density for the ITB formation, a shot-to-shot basis density scan is performed in deuterium (D) dominant plasmas, hydrogen (H) dominant plasmas, and D-H mixed plasmas. Here, the D content determined by a passive spectroscopy is maintained approximately constant at 89 %, 4 %, and 60 %, respectively, during the density scan. Here, the deuterium content is determined as $I_{D_\alpha }/(I_{D_\alpha }+I_{H_\alpha })$, where $I_{D_\alpha }$ and $I_{H_\alpha }$ are the intensities of the $D_\alpha$ emission and the $H_\alpha$ emission measured by a passive spectroscopy, respectively.

For the cases with the D content of 89 % and 4 %, NB source gas and pre-conditioning gas for the plasma facing components (PFCs) are matched to the fueling gas in order to enhance either D or H purity. In such a case, the plasma density is controllable only by the gas puff fueling. For the D-H mixed case, H gas puff fueling is applied with the NB source gas and the PFC pre-conditioning gas of D. Aiming at maintaining the desired mixture rate, extra PFC conditioning discharges with H puff fueling are performed between shots. After such conditioning discharges, precise density control becomes difficult because of a high recycling rate, which results in a limited number of data points in the density scan, as shown below. It is also challenging to perform the D content scan with higher resolution because of the incompatibility between the D content control and the density control.

### Hydrogen isotope effect in ITB threshold

**Figure 1:**
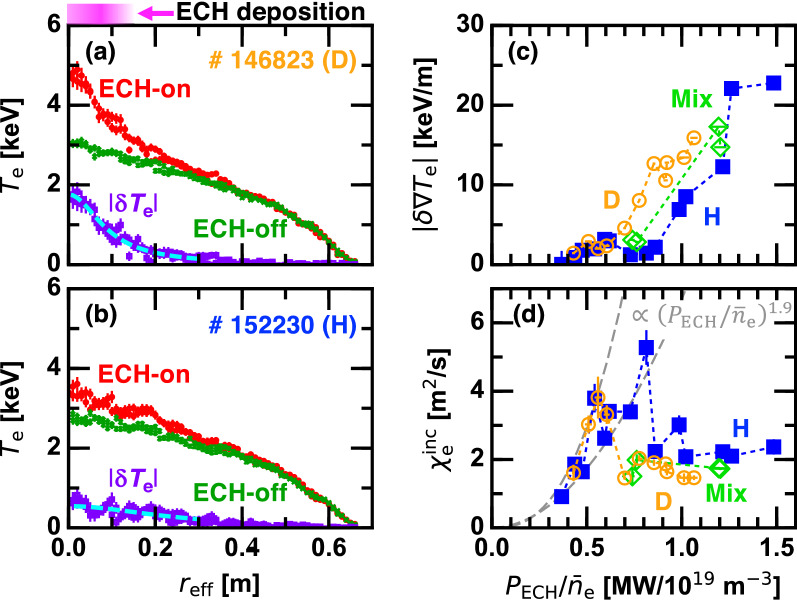
Radial profiles of the electron temperature when the ECH is applied and not applied for (**a**) deuterium plasmas and (**b**) hydrogen plasmas; (**c**) the peak-to-peak modulation amplitude of the electron temperature gradient and (**d**) the incremental heat diffusivity as a function of the applied ECH power normalized by the line averaged density. Magnitude of the profile variation $|\delta T_{\mathrm{e}}|$ defined as difference between the ECH-on and ECH-off profiles is plotted by purple symbols with the Lorentzian fit in (**a**) and (**b**).

Evolution of the electron temperature ($T_{\mathrm{e}}$) profile is measured by the Thomson scattering system^12^. Figure 1a,b compare $T_{\mathrm{e}}$ profiles in the ECH-on and -off phases in D and H discharges at the line averaged density of ${\bar{n}}_{\mathrm{e}} \sim 1.2 \times 10^{19}$ $\hbox {m}^{-3}$. Here, $r_{\mathrm{eff}}$ is the effective minor radius of the torus. In the D plasma (Fig. 1a), central $T_{\mathrm{e}}$ drastically increases when the ECH is applied while the H plasma shows a marginal increment (Fig. 1b). The profile variation $|\delta T_{\mathrm{e}}|$ defined as the difference between the ECH-on and ECH-off profiles is fitted by the Lorentzian function $A\Delta r_{\mathrm{eff}} \left[ r_{\mathrm{eff}}^2+\Delta r_{\mathrm{eff}}^2\right] ^{-1}$, where *A* and $\Delta r_{\mathrm{eff}}$ are fitting parameters, as shown by dashed curves in Fig. 1a,b. The peak-to-peak modulation amplitude in $T_{\mathrm{e}}$ gradient is given as the radial derivative of the Lorentzian fitting function at the inflection points, i.e., $|\delta \nabla T_{\mathrm{e}}| \sim -0.65 \times A \Delta r_{\mathrm{eff}}^{-2}$, and is plotted as a function of the applied ECH power normalized by the line averaged density $P_{\mathrm{ECH}}/{\bar{n}}_{\mathrm{e}}$ in Fig. 1c. When $P_{\mathrm{ECH}}/{\bar{n}}_{\mathrm{e}}$ is low, $|\delta \nabla T_{\mathrm{e}}|$ remains low as well. As $P_{\mathrm{ECH}}/{\bar{n}}_{\mathrm{e}}$ is increased, $|\delta \nabla T_{\mathrm{e}}|$ begins to monotonically increase once $P_{\mathrm{ECH}}/{\bar{n}}_{\mathrm{e}}$ exceeds threshold values that depend on the D content.

**Figure 2:**
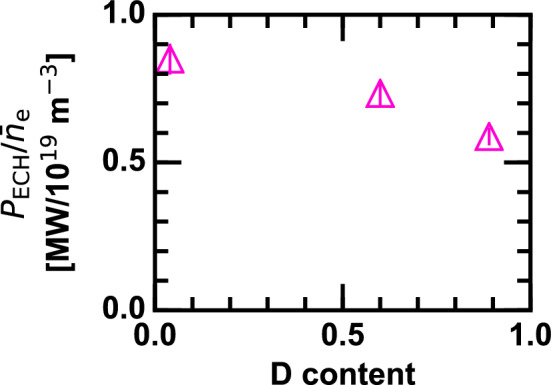
Deuterium content dependence of the threshold value of ECH power normalized by the line averaged density for the ITB transition.

In Fig. 2, the threshold values are plotted as a function of the D content by taking the value of $P_{\mathrm{ECH}}/{\bar{n}}_{\mathrm{e}}$ at which $|\delta \nabla T_{\mathrm{e}}|$ surpasses 2.5 keV/m. Error bars in the threshold diagram is mainly consequent upon an imperfect reproducibility among discharges, i.e., vertical scatters of points in Fig. 1c. The threshold value in $P_{\mathrm{ECH}}/{\bar{n}}_{\mathrm{e}}$ is found to monotonically decay as the D content is increased. Eased threshold condition for the different types of transport barriers with larger ion mass is ubiquitously observed in various torus plasmas including tokamaks and stellarators^4,7–10^, implying existence of an analogous underlying physics possibly related to the radial electric field drive. Note that the energy flow from electrons to ions is less than 40 kW, which is negligibly small and plays a minor role for the threshold condition.

Local transport property is examined through the electron thermal diffusivity obtained in a perturbative manner, the so-called incremental diffusivity $\chi _{\mathrm{e}}^{\mathrm{inc}}$ $\equiv |\delta q_{\mathrm{e}}|/n_{\mathrm{e}}|\delta \nabla T_{\mathrm{e}}|$, where $n_{\mathrm{e}}$ is the local electron density. The electron heat flux variation $\delta q_{\mathrm{e}}$ is given by the energy conservation equation in the ECH-on phase as $\delta q_{\mathrm{e}}=V'^{-1} \int _0^r \left[ p - 1.5 n_{\mathrm{e}} \partial \delta T_{\mathrm{e}}/\partial t \right] V' dr$, where $V'$ is the radial derivative of the plasma volume and *p* is the calculated power deposition profile^13^. The nonlocal part of the electron heat flux (transient variation in $\delta q_{\mathrm{e}}$ immediately after the ECH turn-on or -off) was almost insensitive to the D content^13^. Figure 1d displays $P_{\mathrm{ECH}}/{\bar{n}}_{\mathrm{e}}$ dependence of $\chi _{\mathrm{e}}^{\mathrm{inc}}$ at $r_{\mathrm{eff}}=0.15$ m. In the lower $P_{\mathrm{ECH}}/{\bar{n}}_{\mathrm{e}}$ regime, $\chi _{\mathrm{e}}^{\mathrm{inc}}$ increases with $P_{\mathrm{ECH}}/{\bar{n}}_{\mathrm{e}}$. The upper and lower envelopes of the data points are found to be proportional to $(P_{\mathrm{ECH}}/{\bar{n}}_{\mathrm{e}})^{1.9}$, as shown by grey dashed curves. Gradual confinement degradation as the density is lowered is a common feature of stellarators, and, for instance, is seen in the energy confinement time scaling^14^. In the larger $P_{\mathrm{ECH}}/{\bar{n}}_{\mathrm{e}}$ regime, $\chi _{\mathrm{e}}^{\mathrm{inc}}$ stays off from the $(P_{\mathrm{ECH}}/{\bar{n}}_{\mathrm{e}})^{1.9}$ trend, and is maintained low. Those two regimes correspond to non-ITB and ITB regimes, respectively, and the transition threshold value of $P_{\mathrm{ECH}}/{\bar{n}}_{\mathrm{e}}$ clearly depends on the D content, as discussed above.

### Electrostatic potential measurement by HIBP

**Figure 3:**
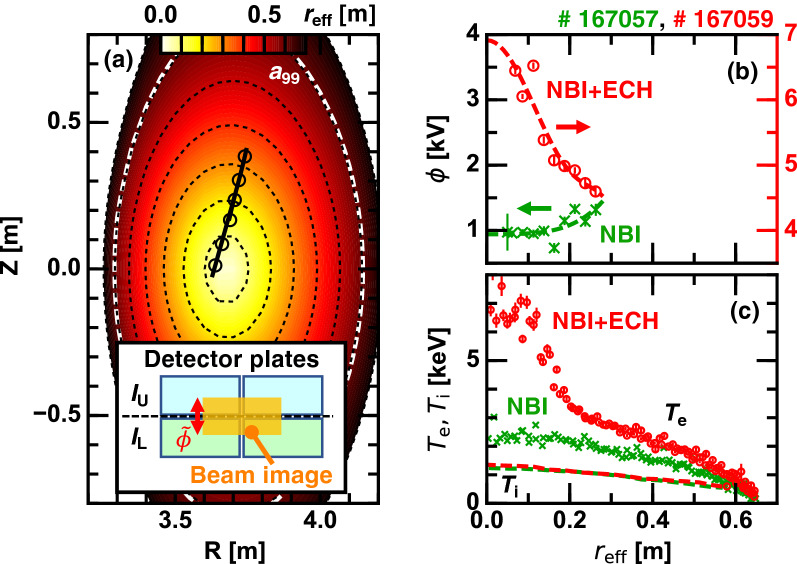
(**a**) Configuration of HIBP projected on the vertically-elongated poloidal cross-section; radial profiles of (**b**) the electrostatic potential and (**c**) the electron and ion temperatures; and (insert) schematic of the HIBP detector plates.

The electron ITB in stellarators is frequently accompanied by a localized positive radial electric field ($E_r$) structure at the core^15–18^, which is primarily induced through the neoclassical process. Therefore, the electron ITB in stellarators is sometimes referred to as the core electron root confinement (CERC). In order to investigate the role of $E_r$ on the isotope effect in the electron ITB threshold, direct measurement of the electrostatic potential ($\phi$) at the plasma core is performed by means of HIBP^19^. HIBP in LHD provides a single point measurement. To obtain the radial profile, the measurement position is scanned either in the 10 Hz reciprocation manner or in a shot-to-shot manner, as shown by the thick curve or the open circles in Fig. 3a, respectively. Here, $a_{99} \sim 0.6$ m is the plasma edge in which 99 % of the electron kinetic energy is confined. The local electrostatic potential is translated into the probe beam image position on detector plates (Fig. 3a insert), which are located in the energy analyzer. Current flowing through the lower and upper detector plates, $I_{\mathrm{L}}$ and $I_{\mathrm{U}}$, respectively, are recorded as the raw HIBP data. The electrostatic potential is given as $\phi = C I_{\mathrm{diff}} I_{\mathrm{HIBP}}^{-1}$, where $I_{\mathrm{diff}}=I_{\mathrm{L}}-I_{\mathrm{U}}$ and $I_{\mathrm{HIBP}}=I_{\mathrm{L}}+I_{\mathrm{U}}$ are the HIBP difference current and the HIBP total current, respectively, and *C* is the proportionality factor.

As an example, radial profile of $\phi$ measured by HIBP in low density (${\bar{n}}_{\mathrm{e}} \sim 0.8 \times 10^{19}$ $\hbox {m}^{-3}$) stationary hydrogen discharges is shown. Figure 3b shows $\phi$ profiles in a balanced NB discharge and a stationary ECH superposed discharge measured by a reciprocation position scan. Figure 3c exhibits the electron and ion temperature profiles, where the electron ITB is formed in the ECH superposed discharge. It is evident that $\phi$ profile with a strong negative gradient, i.e., positive $E_r$, emerges in the core when the ITB is formed. This positive $E_r$ is likely ascribed to the electron root solution in the neoclassical transport balance as described in^15–18^. Meanwhile, the non-ITB plasma has an almost flat $\phi$ profile in the core, which corresponds to the ion root solution.

### Hydrogen isotope effect in core electrostatic potential response

**Figure 4:**
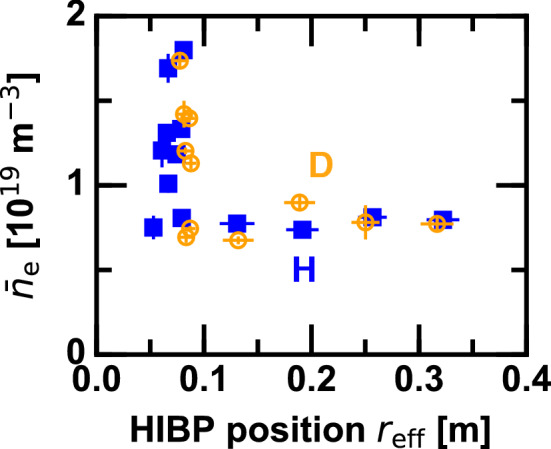
Parameter map of line averaged density and HIBP position on which discharges were performed by a shot-to-shot manner.

**Figure 5:**
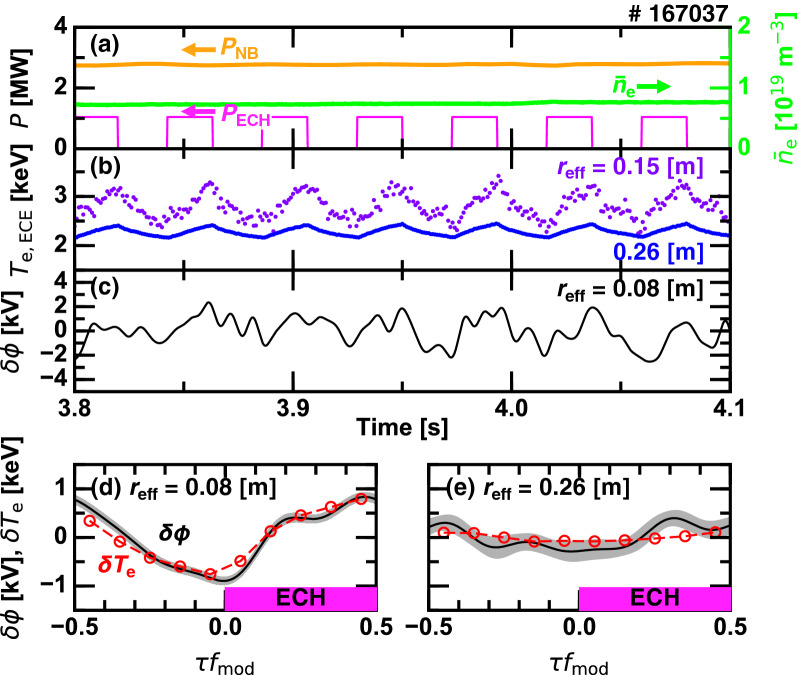
Time evolutions of (**a**) the heating powers and the line averaged electron density, (**b**) the electron temperature, and (**c**) the core electrostatic potential with a 100 Hz low-pass filter; and conditionally averaged electrostatic potential and electron temperature (**d**) inside and (**e**) outside the ITB.

HIBP measurement is applied to the ECH modulation discharge to study the critical behavior in $E_r$ across the ITB formation. Unlike the case shown above, the HIBP position is fixed during a discharge. In order to obtain the radial profile of the $\phi$ modulation amplitude, the HIBP position is scanned in a shot-to-shot manner at a fixed low density condition. While, to explore the electrostatic potential response at the core across the ITB transition, the line averaged density is scanned during which the HIBP position is fixed to the core. Parameter map on which discharges were performed is shown in Fig. 4.

As an example of this dataset, Fig. 5 illustrates the time evolution of a low density hydrogen target discharge. The ECH modulation is applied on the nearly stationary plasma as shown in Fig. 5a. Figure 5b shows the time evolutions of $T_{\mathrm{e}}$ inside ($r_{\mathrm{eff}}=0.15$ m) and outside ($r_{\mathrm{eff}}=0.26$ m) the ITB measured by ECE radiometers. Modulation amplitude drastically varies across the ITB, corresponding to the localized confinement improvement. Oscillations synchronizing with the 23 Hz ECH modulation are also observed in $\phi$ variation in the core region (Fig. 5c). In order to extract the repetitive nature of $\phi$ variation, the conditional averaging is performed as ${\hat{\phi }}(\tau ) = N^{-1} \sum ^{N}_{i=1} \phi (t_i + \tau )$, where $|\tau | < T/2$ and $T = f_{\mathrm{mod}}^{-1}$ is the period of the ECH modulation. The value $t_i$ indicates the *i*-th turn-on time of ECH and *N* is the total number of the modulation. Figure 5d,e show the conditionally averaged $\phi$ signals at $r_{\mathrm{eff}}=0.08$ m and 0.26 m, i.e., inside and outside the ITB, respectively. In addition, reconstructed $T_{\mathrm{e}}$ variation measured by the Thomson scattering system^20^ are overlaid. Inside the ITB, $\phi$ and $T_{\mathrm{e}}$ simultaneously increase when the ECH is applied, while outside their modulation amplitudes are much smaller. This oscillation amplitude difference in $\phi$ is indicative of the existence of $E_r$ oscillation that plays a role for the electron ITB formation. It is worthwhile to note that there is an asymmetry in time evolutions of $\phi$ and $T_{\mathrm{e}}$, i.e., a stepwise increase and a nearly linear decay. The ITB begins to form at the moment of the ECH application, and quickly saturates making the step-like waveform. When the ECH is terminated, the ITB slowly weakens and eventually returns to the non-ITB plasma.

**Figure 6:**
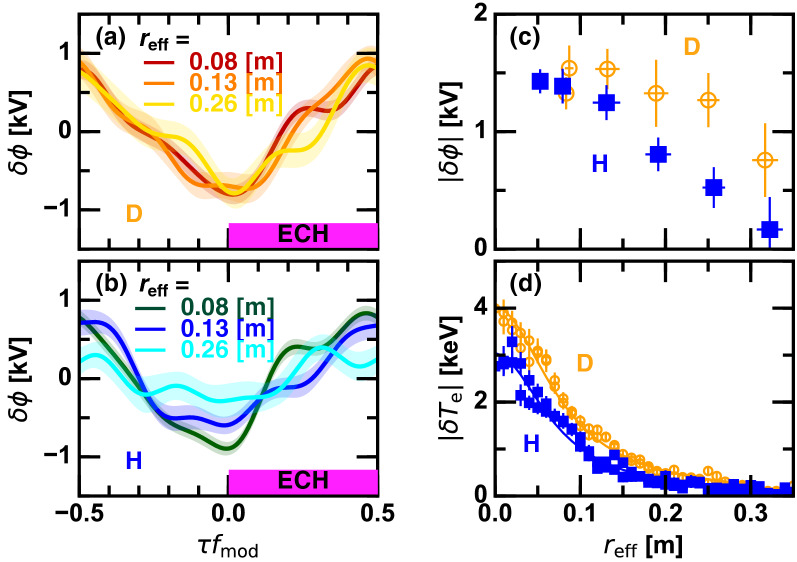
Conditionally averaged electrostatic potentials at various radii for (**a**) deuterium plasmas and (**b**) hydrogen plasmas; and radial profiles of the peak-to-peak modulation amplitude in (**c**) the electrostatic potential and (**d**) the electron temperature.

Figure 6a,b compare the conditionally averaged $\phi$ at different radial positions in D and H low density plasmas of ${\bar{n}}_{\mathrm{e}} \sim 0.8 \times 10^{19}$ $\hbox {m}^{-3}$. The oscillation amplitude gradually decays in the radially outward direction in H plasmas, while it stays nearly unchanged in D plasmas. In order to more distinctly quantify this difference in D and H plasmas, the radial profile of the peak-to-peak $\phi$ oscillation amplitude for the fundamental modulation frequency component (23 Hz) is shown in Fig. 6c. Although the oscillation amplitude is approximately equivalent at the core, its radial extent in D plasmas is wider than that in H plasmas. The $E_r$ peak locations in H and D plasmas are approximately at $r_{\mathrm{eff}} \sim 0.2$ m and $r_{\mathrm{eff}} \sim 0.3$ m, respectively. A stronger but more localized $E_r$ structure at the ITB region is seemingly excited in H plasmas in contrast to a moderate and wider structure peaked at slightly outer radius in D plasmas. Figure 6d shows $T_{\mathrm{e}}$ variation by the ITB formation. The location where the electron temperature gradient modulation is largest is $r_{\mathrm{eff}} \sim 0.1$ m, which is clearly inner than the $E_r$ peak location. D plasmas have a stronger ITB with a slightly wider radial extent. Since $E_r$ is zero in the core by definition, the $E \times B$ flow shear at the ITB location is estimated as $E_r^{\mathrm{peak}}/r_{\mathrm{eff}}^{\mathrm{peak}}$ in the lowest order approximation, where $E_r^{\mathrm{peak}}$ and $r_{\mathrm{eff}}^{\mathrm{peak}}$ are the peak value of $E_r$ and the peak radius, respectively. Therefore, the $E \times B$ flow shear might be stronger in H plasmas due to larger $E_r^{\mathrm{peak}}$ and smaller $r_{\mathrm{eff}}^{\mathrm{peak}}$. Nevertheless, the resultant ITB is stronger in D plasmas, indicating that the $E \times B$ flow shear paradigm is insufficient for describing the observation. Phenomenologically, a wide radial extent of the positive $E_r$ structure and its shear in D plasmas seem to be more effective for transport suppression and significant ITB formation, rather than the large but localized structure in H plasmas. Importance of positive $E_r$ in diminishing the zonal flow damping is argued in^21^, which can be an interpretation of this observation. According to this model, the zonal flow is a hidden key factor, although its detection in the core is still challenging. Enhancement of the zonal flow activity in D plasmas was experimentally addressed in^22^.

**Figure 7:**
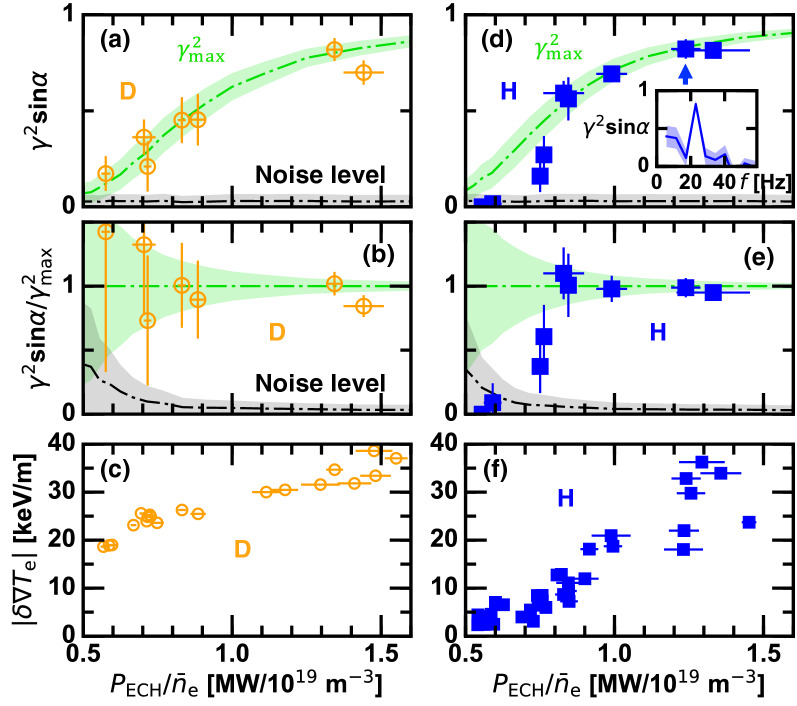
(**a**,**d**) Squared coherence of the HIBP difference current with respect to the ECH modulation pulse, (**b**,**e**) that normalized by its maximum possible value, and (**c**,**f**) the peak-to-peak modulation amplitude of the electron temperature gradient as a function of the applied ECH power normalized by the line averaged density for deuterium plasmas and hydrogen plasmas, respectively; and (insert) squared coherence spectrum for a low density hydrogen discharge.

In investigating the critical role of $E_r$ across the ITB formation, the radial profile of $E_r$ is desirable to be measured in different $P_{\mathrm{ECH}}/{\bar{n}}_{\mathrm{e}}$ settings. However, obtaining those data are allowed only in low density plasmas as shown in Fig. 6, where a high signal to noise ratio is expected. Instead of obtaining the radial profile, the HIBP position is fixed to the core and the line averaged density is scanned in a shot-to-shot basis, as shown in Fig. 4. Once the radial electric field profile is assumed to remain largely unchanged in the electron root regime (such as Figs. 3b and 6c), the $\phi$ modulation amplitude at the plasma core can be a proxy for the integrated magnitude of the positive radial electric field modulation for a qualitative study. Still, a diagnostic difficulty for this experiment remains, which is that the HIBP signal intensity (total current $I_{\mathrm{HIBP}}$) exponentially decays as the density is increased. In such a low signal intensity case, the value of $\phi \propto I_{\mathrm{diff}} I_{\mathrm{HIBP}}^{-1}$ can be uncertain because of small value in the denominator.

In order to settle this problem, the HIBP difference current $I_{\mathrm{diff}}$ is used as a proxy of local $\phi$. Moreover, level of $\phi$ response to the ECH modulation pulse is statistically characterized as $\gamma ^2 \sin \alpha$, where $\gamma ^2$ and $\alpha$ are the squared cross coherence and the cross phase between $I_{\mathrm{diff}}$ and $P_{\mathrm{ECH}}$, respectively. If $\phi$ modulation is induced by the ECH modulation, $\alpha$ must be $\pi /2$ as shown in Figs. 5 and 6. Figure 7a,d show $\gamma ^2 \sin \alpha$ at the modulation ECH frequency of 23 Hz plotted against $P_{\mathrm{ECH}}/{\bar{n}}_{\mathrm{e}}$ for D and H plasmas. An example of the frequency spectrum is given in the insert in Fig. 7d. In both cases $\gamma ^2 \sin \alpha$ decays as $P_{\mathrm{ECH}}/{\bar{n}}_{\mathrm{e}}$ is decreased (${\bar{n}}_{\mathrm{e}}$ is increased).

The decay in $\gamma ^2$ can be caused either due to disappearance of $\phi$ modulation in the core or due to deteriorated signal-to-noise ratio. The latter possibility is examined through a synthetic data approach. Decay characteristic of $\gamma ^2$ is estimated using a mimic HIBP difference current $I_{\mathrm{HIBP}} \equiv {\hat{I}}_{\mathrm{HIBP}} \sin 2 \pi f_{\mathrm{mod}} t+\mathrm{WGN}(\sigma _0)$, where $\mathrm{WGN}(\sigma _0)$ is the white Gaussian noise with a standard deviation of $\sigma _0$. Note that $\sigma _0$ corresponds to the characteristic magnitude of the noise, and is maintained in this examination. The modulation amplitude ${\hat{I}}_{\mathrm{HIBP}}$ is decayed following the known density dependence, ${\hat{I}}_{\mathrm{HIBP}} = I_0 \exp [-n_{\mathrm{e}}/0.55 \times 10^{19}]$, obtained by a dedicated hardware test. The ratio between $I_0$ and $\sigma _0$ is determined in such a way that the $\gamma ^2$ curve passes through the experimental data points in the low density domain, where $\phi$ oscillation is unambiguously observed. The synthetically derived squared cross coherence, referred to as $\gamma ^2_{\mathrm{max}}$, represents the case where the coherence decays only due to the descent in the HIBP signal intensity.

As shown in Fig. 7a, the measured value follows $\gamma ^2_{\mathrm{max}}$ in the entire $P_{\mathrm{ECH}}/{\bar{n}}_{\mathrm{e}}$ range in D plasmas. This indicates that $\phi$ modulation occurs in all the cases in D plasmas and the variation in the coherence is solely due to the declined HIBP signal intensity in higher density range. In contrast, the measured value stays off from the $\gamma ^2_{\mathrm{max}}$ curve at $P_{\mathrm{ECH}}/{\bar{n}}_{\mathrm{e}} \sim 0.8$ MW/10$^{19}$ $\hbox {m}^{-3}$ in H plasmas as displayed in Fig. 7d. It turns out that $\phi$ at the core scarcely responds to the ECH pulse in $P_{\mathrm{ECH}}/{\bar{n}}_{\mathrm{e}} < 0.7$ MW/10$^{19}$ $\hbox {m}^{-3}$. This difference in D and H plasmas is more evident in the relative plot to the synthetic data, $\gamma ^2 \sin \alpha / \gamma ^2_{\mathrm{max}}$, as shown in Fig. 7b,e. Although the error bar is large, the $\phi$ modulation most probably exists in D plasmas even in the low value of $P_{\mathrm{ECH}}/{\bar{n}}_{\mathrm{e}}$, where the $\phi$ modulation is clearly below the noise level in H plasmas. Figure 7c,f indicate the peak-to-peak modulation amplitude in $T_{\mathrm{e}}$ gradient. H plasmas begin to form the ITB structure at $P_{\mathrm{ECH}}/{\bar{n}}_{\mathrm{e}} \sim 0.8$ MW/10$^{19}$ $\hbox {m}^{-3}$, where $\phi$ oscillation becomes substantial. In contrast, the ITB in D plasmas is maintained in the entire range of $P_{\mathrm{ECH}}/{\bar{n}}_{\mathrm{e}}$ with the meaningful $\phi$ oscillation. This observation indicates that the critical behavior in the $E_r$ structure formation can have an isotope effect as well. Considering a leading role of $E_r$ structure for the confinement improvement across the ITB formation^15–17^, the susceptibility of $E_r$ on the plasma ion mass could explain the isotope dependence of the ITB threshold. Note that the threshold value in $P_{\mathrm{ECH}}/{\bar{n}}_{\mathrm{e}}$ is not perfectly identical to the case shown in Fig. 1 due to different experimental conditions.

## Discussion

**Figure 8:**
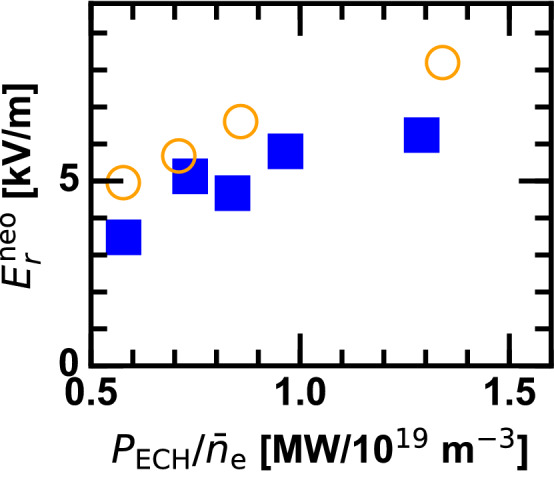
Numerically predicted neoclassical ambipolar radial electric field in the ECH applied phase.

Since the neoclassical $E_r$ transition plays a role for the ITB criticality^15–18^, whether neoclassical $E_r$ has a susceptibility to the plasma ion mass is numerically examined by a local neoclassical transport code DKES/PENTA^23^. Figure 8 shows the $P_{\mathrm{ECH}}/{\bar{n}}_{\mathrm{e}}$ dependence of calculated neoclassical $E_r$ at $r_{\mathrm{eff}} \sim 0.2$ m in the ECH applied phase. Although neoclassical $E_r$ is systematically larger in D plasmas in the electron-root solution, critical behavior is visible neither in D nor H plasmas. This indicates that the isotope effect in the ITB threshold is better explained by the neoclassical theory combined with some additional factors. For example, zonal flows^21^ having an isotopic mass dependence^24^, and likely enhanced in the electron root core with the positive radial electric field, can be a candidate as the underlying mechanism. Global neoclassical effects are also important particularly close to the magnetic axis, which is dependent on the ion gyroradius. Further details about comparison between experiment and simulation are studied in future.

An extra input power necessary for the $E_r$ structure formation at the high-confinement mode transition in hydrogen plasmas is reported in the ASDEX-Upgrade tokamak^9^, which possibly has a link to the present case. In addition, an important role of the fast ions produced by NBs on core transport suppression is recently shown to have an isotope effect^25^, which can also contribute to the isotope effect in the ITB criticality.

## Summary

In conclusion, the isotope effect in the electron ITB threshold favorable to plasmas with heavier ions was found. Across the ITB formation, a transition of the core electrostatic potential, likely leading to a positive radial electric field structure formation, was observed to have the isotope effect as well. A better core plasma performance with ITB in future deuterium-tritium plasmas is therefore foreseen. Transport barrier structures are regarded as a dissipative structure formed spontaneously in nonequilibrium open system by an intensive power input^26^. The present work also contributes to understanding of the dissipative structure affected by basic properties of the media.

## Methods

### Large Helical Device (LHD)

LHD is a plasma current-free magnetically confined torus plasma device. High-temperature plasmas are confined in the externally excited helical magnetic field structure with the toroidal magnetic field of $B_{\mathrm{t}} = 2.75$ T and the vacuum torus axis of $R = 3.6$ m. In the vacuum magnetic field configuration, the rotational transform, $\iota /2\pi = 1/q$, where $q$ is the safety factor, monotonically increases with the radius. The rational surfaces of $\iota /2\pi = 0.5$ and 1 typically exist at the core and the edge, respectively. The LHD data can be accessed from the LHD data repository at https://www-lhd.nifs.ac.jp/pub/Repository_en.html.

### Heavy Ion Beam Probe (HIBP)

HIBP provides a direct measurement of the plasma electrostatic potential^19^. Heavy ion beam injected to the plasma is secondary ionized, where the beam trajectory diverges from the original beam trajectory. A part of the secondary ionized beam is selected by a slit, and the beam energy is analyzed. The energy difference between the original beam and the secondary ionized beam is equivalent to the electrostatic potential at which the secondary ionization occurs. To manipulate the measurement location, operational parameters for the HIBP, such as the injection beam energy, the incident angle, and the confinement magnetic field, are chosen according to the result of the beam trajectory calculation.

## References

[1] Bessenrodt-Weberpals M (1993). The isotope effect in ASDEX. Nucl. Fusion.

[2] Hawryluk RJ (1998). Results from deuterium-tritium tokamak confinement experiments. Rev. Mod. Phys..

[3] Cordey JG (1999). Plasma confinement in JET H mode plasmas with H, D, DT and T isotopes. Nucl. Fusion.

[4] Maggi CF (2018). Isotope effects on LH threshold and confinement in tokamak plasmas. Plasma Phys. Control. Fusion.

[5] Yamada H (2019). Isotope effect on energy confinement time and thermal transport in neutral-beam-heated Stellarator–Heliotron Plasmas. Phys. Rev. Lett..

[6] Miyamoto K (2004). Plasma physics and controlled nuclear fusion, Chapter 7.

[7] Yan Z (2017). Turbulence and sheared flow structures behind the isotopic dependence of the LH power threshold on DIII-D. Nucl. Fusion.

[8] Plank U (2020). H-mode power threshold studies in mixed ion species plasmas at ASDEX Upgrade. Nucl. Fusion.

[9] Cavedon M (2020). Connecting the global H-mode power threshold to the local radial electric field at ASDEX Upgrade. Nucl. Fusion.

[10] Kobayashi T (2019). Isotope effects in self-organization of internal transport barrier and concomitant edge confinement degradation in steady-state LHD plasmas. Sci. Rep..

[11] Kobayashi T (2020). The physics of the mean and oscillating radial electric field in the L-H transition: The driving nature and turbulent transport suppression mechanism. Nucl. Fusion.

[12] Yamada I (2010). Recent progress of the LHD Thomson scattering system. Fus. Sci. Technol..

[13] Kobayashi T (2020). Isotope effect in transient electron thermal transport property and its impact on the electron internal transport barrier formation in LHD. Nucl. Fusion.

[14] Yamada H (2005). Characterization of energy confinement in net-current free plasmas using the extended International Stellarator Database. Nucl. Fusion.

[15] Fujisawa A (1999). Electron thermal transport barrier and density fluctuation reduction in a toroidal helical plasma. Phys. Rev. Lett..

[16] Stroth U (2001). Internal transport barrier triggered by neoclassical transport in W7-AS. Phys. Rev. Lett..

[17] Estrada T (2003). Electron internal transport barrier formation and dynamics in the plasma core of the TJ-II stellarator. Plasma Phys. Control. Fusion.

[18] Ida K (2003). Characteristics of electron heat transport of plasma with an electron internal-transport barrier in the large helical device. Phys. Rev. Lett..

[19] Ido T (2006). 6 MeV heavy ion beam probe on the Large Helical Device. Rev. Sci. Instrum..

[20] Kobayashi T (2016). Reconstruction of high temporal resolution Thomson scattering data during a modulated electron cyclotron resonance heating using conditional averaging. Rev. Sci. Instrum..

[21] Itoh K (2007). Physics of internal transport barrier of toroidal helical plasmas. Phys. Plasmas.

[22] Xu Y (2013). Isotope effect and multiscale physics in fusion plasmas. Phys. Rev. Lett..

[23] Spong DA (2005). Generation and damping of neoclassical plasma flows in stellarators. Phys. Plasmas.

[24] Hahm TS (2013). Isotopic dependence of residual zonal flows. Nucl. Fusion.

[25] Schneider PA (2021). Fast-ion pressure dominating the mass dependence of the core heat transport in ASDEX Upgrade H-modes. Nucl. Fusion.

[26] Razumova KA (2011). Tokamak plasma self-organization-synergetics of magnetic trap plasmas. Nucl. Fusion.

